# Current management practices and interventions prioritised as part of a nationwide mastitis control plan

**DOI:** 10.1136/vr.103203

**Published:** 2016-03-10

**Authors:** P. M. Down, A. J. Bradley, J. E. Breen, C. D. Hudson, M. J. Green

**Affiliations:** 1 University of Nottingham, School of Veterinary Medicine & Science, Sutton Bonington Campus, Sutton Bonington, Loughborough, LE12 5RD, UK; 2 Quality Milk Management Services Ltd, Cedar Barn, Easton Hill, Easton, Wells BA5 1DU, UK

**Keywords:** Herd health, Dairy Cow, Mastitis control, AHDB Dairy Mastitis Control Plan

## Abstract

The objectives of this study were to report performance and management data taken from a sample of UK dairy farms that have participated in the Agriculture and Horticulture Development Board Dairy Mastitis Control Plan (DMCP) and to identify important mastitis prevention practices that are not currently widely implemented. A total of 234 UK dairy herds were included in the study from which farm management and udder health data were collected. Herds were grouped according to their mastitis epidemiology and could be classed as (i) environmental dry period (EDP) (i.e. environmental pathogen with majority of infections being acquired during the dry period), (ii) environmental lactation (EL), (iii) contagious dry period (CDP) or (iv) contagious lactation (CL). The results of this study showed that many mastitis-related management practices that are generally considered to be important were not widely performed. A better understanding of those practices not widely adopted by UK dairy farmers at present may aid practitioners in identifying and overcoming potential barriers to improved mastitis control.

## Introduction

MASTITIS remains one of the most significant diseases affecting dairy cows in the UK and worldwide resulting in large economic losses to the industry and compromised welfare for the cows affected (Bradley 2002, Halasa and others 2007). Despite an ever-growing body of research into the risk factors and epidemiology of intramammary infections, the incidence of mastitis in UK dairy herds has remained fairly static during recent decades (Booth 1988, Kossaibati and others 1998, Bradley and Green 2001). The most recent large-scale study estimated the current mean herd incidence to be between 47 and 65 cases per 100 cows per year in the UK (Bradley and others 2007a).

A detailed mastitis control plan was devised and tested in a randomised controlled trial during 2004–2005 and found to reduce clinical mastitis (CM) and new infections as measured by somatic cell count (SCC) by approximately 20 per cent (Green and others 2007a). It was subsequently rolled out as a national scheme in 2009 as the Agriculture and Horticulture Development Board (AHDB) Dairy Mastitis Control Plan (DMCP) (Bradley and others 2009). The DMCP is delivered by trained veterinarians and consultants and involves data analysis, a detailed questionnaire and on-farm observations and measurements. Using this information, farm-specific recommendations are prioritised for discussion with the farmer with the aim of implementing changes most relevant to the underlying epidemiology of mastitis on the farm. Since the DMCP was launched in 2009, over 300 plan deliverers have been trained and over 2000 farms have had some involvement with the plan, representing approximately 20 per cent of the national herd. Since the scheme began, the national bulk milk somatic cell count (BMSCC) has fallen by 15 per cent over a five-year period (DairyCo 2015).

A variety of studies have considered on-farm management practices relevant to mastitis control but there have been relatively few peer-reviewed studies from the UK (Fenlon and others 1995, Green and others 2007b, Green and others 2008, Langford and others 2009) and nothing as detailed as the DMCP questionnaire which has 377 questions and observations all relevant to mastitis control and prevention. A better appreciation of current management practices would aid the understanding of why mastitis remains such a problem on many UK dairy farms and provide useful insights into which interventions are perceived to be most important for different types of farms. The purposes of this study were to report performance and management data taken from a sample of UK dairy farms that have participated in the DMCP and to identify important mastitis prevention practices that are not currently widely implemented by farmers. The frequency at which these deficiencies in management were prioritised by the plan deliverers was also reported to evaluate how important these management practices were perceived to be by vets.

## Materials and methods

### AHDB DMCP

Farms typically participated in the DMCP if they were concerned about udder health on their farm or their veterinarian had highlighted mastitis as an area for improvement as part of an ongoing herd health service. The plan would usually be delivered by the farm's own veterinary practice or by a dairy consultant. Veterinary practices could alternatively arrange for an external plan deliverer to implement the plan if they were not able to deliver it directly.

The first stage of conducting the plan on the farm involved detailed data analysis whereby the mastitis epidemiology was described according to the likely mode of pathogen transmission involved (environmental or contagious) and the stage of lactation at which new infections were most often occurring as well as seasonality, parity and recurrence. A categorical herd ‘diagnosis’ was made based on this (Green and others 2007b) and could be either environmental dry period (EDP) (i.e. environmental pathogen with majority of infections being acquired during the dry period), environmental lactation (EL), contagious dry period (CDP) or contagious lactation (CL); this is a required first step of the plan.

The next stage involved a farm visit during which a comprehensive questionnaire was completed consisting of questions for the farmer and observations/measurements made by the plan deliverer. The questionnaire contained 377 questions in 12 sections consisting of general farm information, milking herd management between milkings, premilking management, milking routine, milking plant, postmilking management, management of the dry period, calving cows, treatment of mastitis, biosecurity, dairy replacements and mastitis monitoring. The answers to the questionnaire and the ‘diagnosis’ made were entered into a software package called the ‘DMCP ePlan’ (SUM-IT Computer Systems Ltd 2009). Once all of the required information was entered, the programme would identify where the herd differed from ‘best practice’ in terms of mastitis control and highlight specific interventions most relevant to the herd diagnosis. For example, a lack of premilking teat disinfection would only be highlighted if the herd had an EL diagnosis.

The plan deliverer would prioritise 5–10 of these interventions to be implemented on the farm. A three-level ranking system was used for the interventions based on the strength of evidence from research to assist the plan deliverer in prioritising which interventions to focus on; interventions supported by most evidence were made the priority for action (DairyCo 2014).

### Farm selection

Participating farms were included in this study if the herd performance data (e.g. SCC data and CM records) were available at the plan start date in addition to the ePlan data (the answers to the questionnaire, the herd diagnosis and the prioritised interventions).

### Data collection

Herd performance data were submitted electronically by the plan deliverer when each farm was enrolled on the DMCP. Plan deliverers were contacted directly by the author and asked to send relevant ePlan data.

The non-invasive nature of this project and anonymised data collection meant that formal ethical committee approval was not necessary.

### Data analysis

The herd performance and ePlan data were imported into Microsoft Access, checked and exported into Microsoft Excel for analysis. The herds were grouped accordingly for analysis: EDP, EL and CDP/CL. The CDP/CL herds were grouped together due to similarities in the epidemiology and low numbers of herds assigned those contagious diagnoses.

The parameters used to measure mastitis performance in the participating herds are defined in Table 1 and were BMSCC (12-month mean calculated from individual cow SCCs weighted for milk production), incidence of clinical mastitis (IRCM, cases/100 cows/year), new lactation origin infection incidence/month, as measured by SCC (LNIR) and CM records (clinical mastitis of lactation origin rate (CML)) and new dry period infection incidence/month, as measured by SCC (DPNIR) and CM records (clinical mastitis of dry period origin rate (CMDP)) (Bradley and others 2007a, 2008). Mann-Whitney-Wilcoxon tests were used to compare the 12-month means of the mastitis parameters between the three groups of herds and a significance probability was set at P≤0.05 for a two-tailed test.

**TABLE 1:** Mastitis parameter definitions*

Mastitis parameter	Definition
Lactation new infection rate (LNIR)	The percentage of ‘uninfected’ cows (<200,000 cells/ml for the whole of the current lactation or <200,000 cells/ml at the previous three milk recordings or below 100,000 cells/ml at the previous two milk recordings if previously >200,000 cells/ml in this lactation) that crossed the 200,000 cells/ml threshold at the following milk recording (target <5 per cent per month).
Dry period new infection rate (DPNIR)	The percentage of cows (and heifers) ‘infected’ (>200,000 cells/ml*) in the first 30 days after calving that were ‘uninfected’ (<200,000 cells/ml) in the milk recording within 1 month of drying-off (target <10 per cent per month) (*>400,000 cells/ml if recorded within five days of calving†).
Dry period cure rate (DPCURE)	The monthly percentage of ‘infected’ cows (>200,000 cells/ml) before drying-off that were ‘uninfected’ (<200,000 cells/ml*) at the first milk recording after calving (*<400,000 cells/ml if recorded within 5 days of calving†).
Clinical mastitis of lactation origin rate (CML)	The incidence of first (index) cases occurring in lactation, 31–305 days in milk (target <2 in 12 cows per lactation period (<16.66 cases/100 cows/year)).
Clinical mastitis of dry period origin rate (CMDP)	The incidence of first (index) cases occurring at <31 days in milk (likely dry period origin) (target <1 in 12 cows per 30-day period (<8.33 cases/100 cows/year)).

*Based on Bradley and others (2007a, 2008)

†Based on Barkema and others (1999a)

The proportion of herds that were not performing each intervention was calculated and the frequency with which each intervention was ‘prioritised’ by the plan deliverers was also calculated. The interventions were ranked according to the proportion of eligible herds that undertook them and the interventions that were least commonly practised were reported (Table 3).

## Results

**FIG 1:**
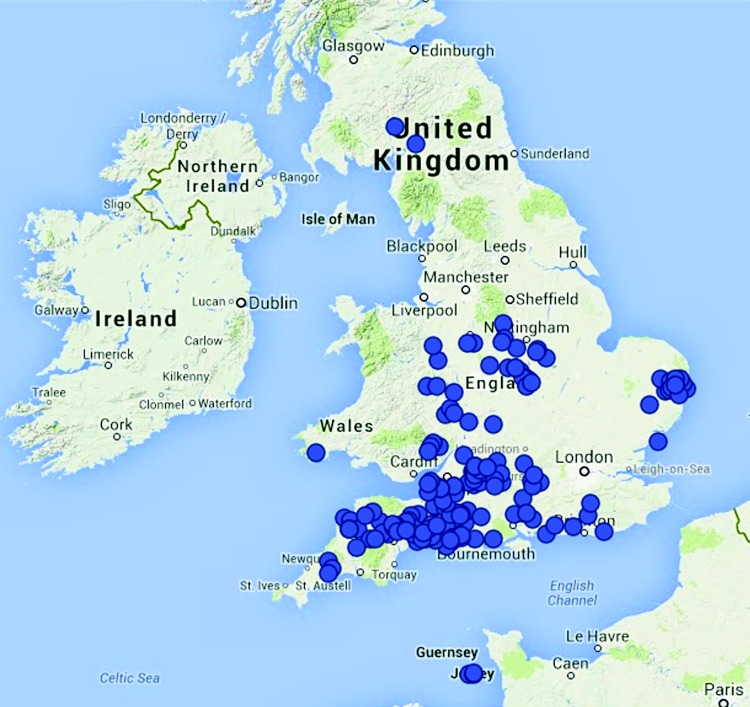
Geographical location of herds in the study

A total of 234 herds were included in the study that had been enrolled on the DMCP between 2009 and 2012. The geographical location of the farms is shown in Fig 1. The median herd size was 184 cows (range 51–973), which is greater than the current UK average of 133 (AHDB Dairy 2014b). The median 305-day milk yield of the 234 herds was 8463 l (4297–12410), which is also greater than the current national average of 7870 (AHDB Dairy 2014a).

### Mastitis parameters

Differences between the mastitis parameters for the different groups of herds are shown in Table 2. The median BMSCC for all herds was 208,000 cells/ml (range 74,000–809,000) and the median IRCM was 57 cases/100 cows/year (range 6–164). The incidence of new lactation origin infections as measured by SCC (LNIR) and clinical mastitis records (CML) was higher for the herds with a CL/CDP and EL diagnosis than farms with an EDP diagnosis. The apparent cure rate during the dry period as measured by SCC (dry period cure rate (DPCURE)) was significantly higher in EL herds than the EDP (P=0.0009) and CL/CDP herds (P=0.003). The incidence of dry period origin infections as measured by CM data (CMDP) was significantly higher in the EDP herds than the herds with an EL (P<0.0001) or CL/CDP (P=0.0002) diagnosis (Table 2).

**TABLE 2:** General performance parameters and mastitis indices

	EDP	EL	CDP/CL	Overall (median)
Number	111	103	20	234
Herd size	51–553 (200)	52–973 (216)	74–390 (176)	51–973 (184)
305 days yield* (l)	4297–10663 (8496)	4770–12410 (8509)	6496–10198 (7997)	4297–12410 (8463)
BMSCC†(×1000 cells/ml)	74–809 (220)	79–670 (221)	91–421(249)	74–809 (208)
IRCM‡ (cases/100 cows/year)	18–164 (65)	6–145 (58)	21–122 (58)	6–164 (63)
LNIR§ (%)	4.1–19.2(8.5)^a^	4.6–20.7 (9.3)	7.3–17.1 (10.4)^b^	4.1–20.7 (8.9)
DPNIR¶ (%)	5.3–38 (19.2)^a^	5.8–50 (15.6)^b^	9.3–32 (18.8)^a^	0–63.6 (17.25)
DPCURE**	46.2–96.1 (72.7)^a^	53.8–92.6(76.8)^b^	44.7–89.1 (68.7)^a^	44.7–96.1 (74.15)
CMDP†† (number of cases per 12 cows/%)	0.61–4.75 (1.82/15.17%)^b^	0.04–2.99 (1.01/8.42%)^a^	0.33–2.2 (1.04/8.67%)^a^	0.04–4.75 (1.36/11.33%)
CML‡‡ (number of cases per 12 cows/%)	0.70–4.94 (2.65/22.08%)^a^	0.34–6.97 (3.09/25.75%)^b^	1.96–4.61 (2.67/22.25%)	0.34–6.97 (2.78/23.17%)

The range of 12-month averages is given (lowest–highest) with median value in parenthesis

*Themean total milk yield/cow during the first 305 days of lactation for the herd

†Bulk milk somatic cell count—calculated from individual cow somatic cell counts weighted for milk production

‡Incidence of clinical mastitis (cases/100 cows/year)

§Lactation new infection rate (the monthly percentage of cows previously <200,000 cells/ml cows crossing the 200,000 cells/ml threshold since the last monthly recording)

¶Dry period new infection rate (the monthly percentage of cows that have been recorded for the first time this lactation and are <31 days in milk that are >200,000 cells/ml and were <200,000 cells/ml at drying-off). Heifers are always assumed to be <200,000 cells/ml before first calving

**Dry period cure rate (the monthly percentage of cows that were recorded >200,000 cells/ml before drying-off that were <200,000 cells/ml at the first recording after calving

††Incidence of first (index) clinical mastitis cases of dry period origin/month (<31 days in milk)

‡‡Incidence of first (index) clinical mastitis cases of putative lactation origin/month (i.e. >30 days in milk)

^a,b^Significantly different within row (P≤0.05)

BMSCC, bulk milk somatic cell count; CDP, contagious dry period; CL, contagious lactation; CMDP, clinical mastitis of dry period origin rate; CML, clinical mastitis of lactation origin rate; DPCURE, dry period cure rate; DPNIR, dry period new infection rate; EDP, environmental dry period; EL, environmental lactation; IRCM, incidence of clinical mastitis; LNIR, lactation new infection rate

### Herd management practices

The interventions that were most frequently found not to be undertaken in herds with different diagnoses are displayed in Table 3. Only those interventions relevant to each diagnosis were included in these results. The frequency at which interventions were prioritised by the plan deliverers is presented in Table 3. The number of interventions prioritised on each farm ranged from 1 to 92 with a median of 22.

**TABLE 3:** Proportion of herds currently practising each intervention at the time of study, proportion of herds not practising each intervention that was prioritised by the plan deliverer and the proportion of herds not practising each intervention that was not prioritised by the plan deliverer (ranked in order of least commonly practised)

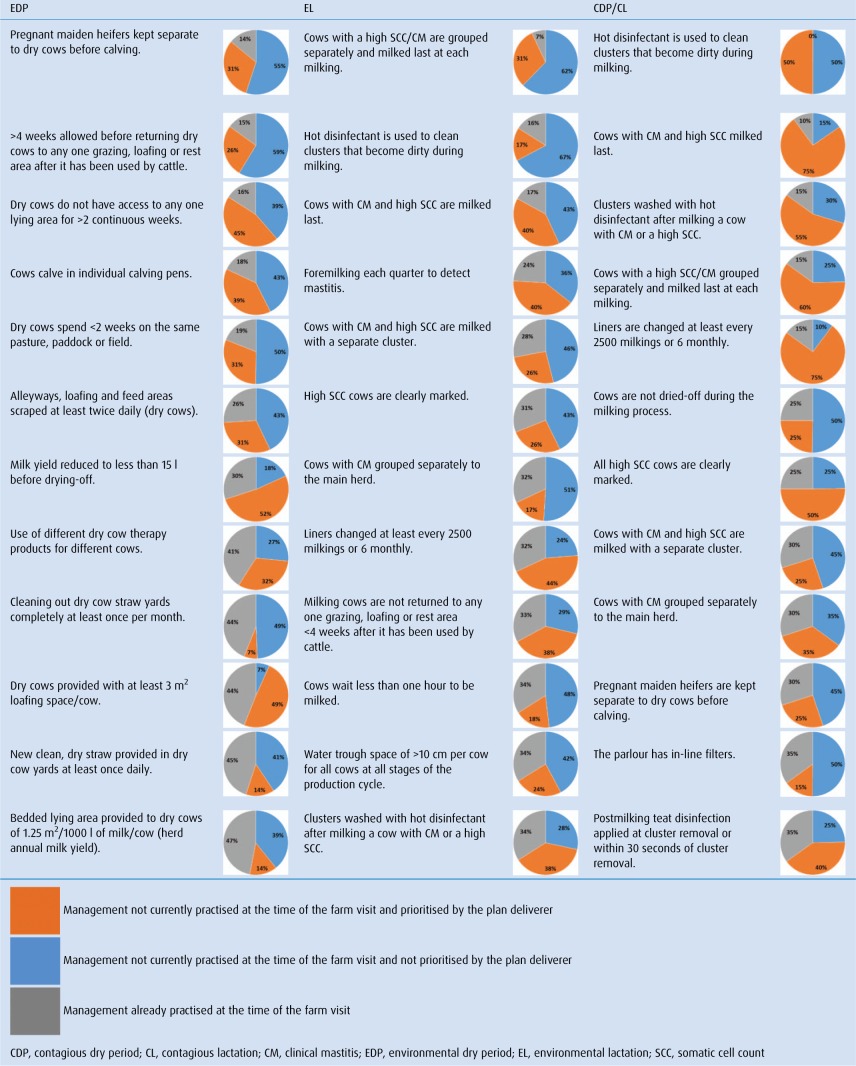

The three least commonly practised interventions in the EDP herds were the separation of heifers from dry cows before calving, allowing at least four weeks before returning dry cows to any one grazing, loafing or rest area after it has been used by cattle and not allowing dry cows to have access to any one lying area for more than two weeks. The three least commonly practised interventions in the EL herds were grouping cows with a high SCC/CM separately and milking them last at each milking, using hot disinfectant to clean clusters that become dirty during milking and milking cows with a high SCC/CM last.

## Discussion

The results of this study show that many mastitis-related management practices that are generally considered to be important were not widely performed in a large sample of UK dairy herds. This is one of the most comprehensive field studies of its kind and the first to group the herds according to the putative origin of new mastitis cases. This grouping is important as the most significant aspects of mastitis control for a CL herd are very different from those for an EDP herd and therefore by grouping herds in this way, the authors are able to highlight the most relevant management ‘deficiencies’.

While representing a relatively large sample of UK dairy herds for this type of study, it is likely that the results are biased towards herds seeking veterinary input with respect to mastitis control rather than being representative of the national herd as a whole. However, this may provide a true reflection of dairy herds seeking veterinary input with respect to mastitis control and therefore is of value to those involved in the delivery of these services. While reasonable steps were taken to ensure all data were accurate and complete, no formal validation of CM data was undertaken.

### EDP herds

Management of the dry cow/calving cow accommodation to maximise hygiene was an area of potential weakness highlighted in this study. Dry cows had continual access to the same pasture/lying area for more than two weeks in over 80 per cent of EDP herds and were allowed to return to paddocks within four weeks of them being previously grazed in 85 per cent of EDP herds. The ‘graze 2, rest 4’ strategy, that is, graze the same paddock for no more than two continual weeks followed by at least a 4-week rest period, has been found to be very effective at reducing the risk of CM in the first 30 days after calving (Green and others 2007b) and was commonly prioritised by the plan deliverers in this study.

The size of the bedded lying area for dry cows was insufficient in over half of the EDP herds in this study despite research demonstrating the importance of this with respect to SCC in the first 30 days of lactation (Green and others 2008). Other practices not undertaken by the majority of EDP herds include adding fresh bedding to the dry cows daily and scraping alleyways, loafing and feed areas twice daily which have been associated with a reduced risk of CM in the first 30 days of lactation (Green and others 2007b). Each of these examples was highly prioritised by the plan deliverers, reflecting the perceived importance associated with dry cow environmental management for these herds.

Less than 20 per cent of the EDP herds used individual calving pens despite evidence that they are associated with a reduced SCC and reduced incidence of CM (Bartlett and others 1992, Barnouin and others 2004, O'Reilly and others 2006). This indicates that many cows are calving in the dry cow yards and almost 60 per cent of EDP herds were failing to completely clean out these straw yards on a monthly basis, which may result in increased CM (Peeler and others 2000). The use of individual calving pens and the cleaning out of dry cow yards were prioritised in 50 per cent and 88 per cent of cases, respectively, once again reflecting the importance of dry period hygiene and possibly reflecting the practical difficulties that come with implementing individual calving pens on some dairy farms.

Almost 60 per cent of the EDP herds were not selecting dry cow therapy (DCT) at cow level in this study (DCT products selected according to the infection status at drying-off) and this was made a priority in 45 per cent of the herds not doing so (Table 3). Whole-herd antibiotic DCT has been recommended as part of the five-point plan for several decades (Neave and others 1969) with the aim of curing existing intramammary infections (IMIs) and preventing new IMIs during this time (Smith and others 1966). There is, however, a growing body of evidence showing potential advantages of selecting DCT at the cow level rather than the herd level due to the impact of total antimicrobial usage on the farm (Scherpenzeel and others 2014) as well as a reduction in CM caused by Gram-negative bacteria (Bradley and others 2010) and a reduced overall risk of CM in the first 30 days of lactation (Green and others 2007b).

Less than 30 per cent of EDP herds were reducing yields to below 15 l before drying-off and this was only prioritised in 26 per cent of cases suggesting that other interventions were deemed more important for most EDP herds. Increased yields at drying-off have been associated with increased SCC (Green and others 2008) and IMI at calving (Dingwell and others 2004, Rajala-Schultz and others 2005, Odensten and others 2007), which is considered to be in part as a result of delayed formation of the keratin plug in the teat due to milk leakage (Dingwell and others 2004). Two strategies employed to reduce the milk yield before drying-off include feed restriction and reduced milking frequency (Bushe and Oliver 1987) and while both are effective, the restriction of feed followed by abrupt cessation of milking was associated with a reduced risk of IMI during the dry period (Tucker and others 2009).

The vast majority (86 per cent) of EDP herds mixed the heifers with the cows before calving. However, several studies have demonstrated that the mixing of maiden heifers and cows during the dry period is associated with increased rates of CM (Barkema and others 1999b) and increased SCC (De Vliegher and others 2004). Recent studies have also shown that heifers which have a raised SCC at the first milk recording post partum are less productive over the whole of their lifetime and have decreased longevity (De Vliegher and others 2005, Piepers and others 2009, Archer and others 2013, 2014), and this is probably why it was made a priority for 64 per cent of these herds.

### EL herds

For herds with an EL diagnosis, key focus areas include the management of the milking cows’ environment as well as the milking routine and machine maintenance. The management of high SCC cows and those with CM featured prominently and it was interesting that ‘infected’ cows were rarely housed separately to the main herd in the present study despite good evidence of the benefits of doing so (Wilson and others 1995, Middleton and others 2001, Zecconi and others 2003). Where this is not possible, it is still preferable to milk these infected cows last but again this was not practised in 83 per cent of the EL herds despite the association with reductions in SCC (Hutton and others 1991, Wilson and others 1995, Barnouin and others 2004). If neither of these approaches is practical, then a pragmatic solution may be to at least mark infected cows so they are easily identifiable and milk them with a separate cluster, but these were also poorly practised despite evidence suggesting an association with reduced SCC (Barnouin and others 2004).

Another aspect of management relating to the hygiene of the milking plant that was not widely practised was the replacement of liners at the appropriate interval. This highlights the value in the AHDB DMCP approach in that it ensures that mastitis control measures that are often assumed to be universally implemented are investigated and rectified when found to be lacking.

The practice of foremilking was only carried out in approximately a quarter of the EL herds in this study. Foremilking is typically recommended to detect CM and is also a means of premilking stimulation (Wagner and Ruegg 2002). The application of foremilking is well established in mastitis control programmes (Rodrigues and others 2005) as it facilitates the rapid detection of CM allowing for the prompt treatment and therefore increased likelihood of successful outcomes (Hillerton and Semmens 1999).

Two-thirds of the EL herds were not following the ‘graze 2, rest 4’ principle as described previously and the same number of herds were allowing cows to wait for more than 1 hour to be milked. These aspects of environmental management could both result in an increased exposure of the cows teats to pathogens in addition to the increased risk of lameness caused by increased waiting times before milking (Espejo and Endres 2007).

### CDP/CL herds

Many of the management practices least implemented by the CDP/CL herds were the same as for the EL herds and focused primarily on the risk of transmission during the milking process as would be expected. Due to the low number of herds with a CDP or CL diagnosis, they were combined as one group for the purposes of this study and the risk factors, therefore, were taken to be the same for both. Perhaps the most striking feature concerning these herds was how few of them grouped or milked cows according to their infection status or replaced the liners at the correct interval which for these herds is likely to be of paramount importance. This was reflected in the high proportion of such interventions that were prioritised by the plan deliverers for the CDP/CL herds.

The majority of herds in this study (87 per cent) were classified as having a predominantly environmental pattern of disease divided almost equally between EDP and EL diagnoses. This was not unexpected as it reflects the national trend for the increased importance of the cows environment as a source of intramammary infections relative to the contagious spread of pathogens from cow to cow that was more common historically (Bradley 2002, Bradley and others 2007a). Contagious pathogens are relatively well controlled by the five-point plan which was introduced in the 1960s and adopted widely by dairy farmers in the UK (Bradley 2002). Unfortunately, this strategy was not designed to control the environmental routes of transmission and so a more farm-specific approach is required to identify risk factors and implement appropriate interventions accordingly.

The importance of the dry period with respect to mastitis control has been well documented (Bradley and Green 2004) and it is known that a significant proportion of CM cases occurring within the first 30 days after calving will have been caused by infections acquired during the dry period (Bradley and Green 2000, Green and others 2002). For herds where these types of infections predominate, the impact that deficiencies in dry cow management may have on udder health and productivity can be profound and should therefore be the focus of any mastitis control plan (Green and others 2007b). Approximately half of the herds in this study were assigned a dry period origin diagnosis and as this is the first large-scale study to categorise herds in this way, it is not possible to say if this is typical of the national population.

The EDP herds had similar BMSCC and CM rates as the other herds in the study but were characterised by a significantly higher rate of CMDP than the other herds when the plan was first implemented as would be expected. They also had a significantly higher rate of DPNIR than the EL herds. Suggested targets for the rate of DPNIR and CMDP are 10 per cent and 1 in 12 (8.33 cases/100 cows/year), respectively, and the averages for the EDP herds in this study were considerably higher than these.

Herds with an EL diagnosis had a similar BMSCC and CM rate to the other herds in the study but were characterised by significantly lower rates of DPNIR and significantly higher DPCURE rates than the other herds as well as a significantly higher rate of CML than the EDP herds. Suggested targets for LNIR and CML are 5 per cent and 2 in 12 (16.66 cases/100 cows/year), respectively.

There were far fewer herds with a ‘contagious’ diagnosis in this study. The CDP/CL herds were characterised by a lower average milk yield than the other herds in the study and a higher BMSCC which would be expected due to the increased chronicity associated with IMIs caused by ‘contagious’ pathogens (Bradley and others 2007b). All other mastitis parameters were broadly similar to the other herds in the study with the exception of the dry period cure rate which was the lowest of all the groups, reflecting the increased challenge of curing infections caused by ‘contagious pathogens’ (Barkema and others 2009).

The frequency with which the interventions reported in this study were prioritised by the plan deliverers varied widely. When interventions were not highly prioritised, this may reflect the presence of more pressing concerns in those particular herds or perhaps a lack of perceived efficacy. It should be recognised that the process of prioritisation would be influenced by a combination of guidance from the ePlan and subjective assessment from the plan deliverer; it is uncertain the extent to which each of these elements influenced the final decisions made on prioritisation. With a limited number of intervention studies from which to draw from, it is very difficult to have much certainty about the efficacy of most mastitis interventions at the individual herd level and any uncertainty about the clinical and financial benefit of an intervention will affect the decision to implement it (Green and others 2010, Huijps and others 2010). A useful continuation of this study would be an investigation into what effect different management interventions or combinations of interventions may have on the mastitis performance for different types of herd, thus facilitating an evidence-based approach to decision making.

## Conclusions

The results of this study provide data on performance and management of UK dairy herds, grouped according to the main putative origin of new cases of mastitis. Many aspects of management that might be considered to be important in mastitis control were not being practised by a large proportion of these herds. A better understanding of those practices not widely adopted by UK dairy farmers at present may aid practitioners in identifying and overcoming potential barriers to improved mastitis control in UK dairy herds.
